# No difference in survival after HLA mismatched versus HLA matched allogeneic stem cell transplantation in Ewing sarcoma patients with advanced disease

**DOI:** 10.1038/s41409-020-01200-x

**Published:** 2021-01-29

**Authors:** U. Thiel, S. J. Schober, A. Ranft, H. Gassmann, S. Jabar, K. Gall, I. von Lüttichau, A. Wawer, E. Koscielniak, M. A. Diaz, M. Ussowicz, I. Kazantsev, B. Afanasyev, M. Merker, T. Klingebiel, A. Prete, B. Gruhn, P. Bader, H. Jürgens, U. Dirksen, R. Handgretinger, S. Burdach, P. Lang

**Affiliations:** 1 Technical University of Munich, School of Medicine, Department of Pediatrics and Children’s Cancer Research Center, Kinderklinik München Schwabing, Munich, Germany; 2 Pediatrics III, West German Cancer Centre Essen, University Hospital Essen, Essen, Germany; 3 Department of Pediatric Oncology, Hematology and Immunology, Olgahospital, Stuttgart, Germany; 4 Department of Pediatric Hematology—Oncology and Hematopoietic Stem Cell Transplantation, Hospital Infantil Universitario Niño Jesus, Madrid, Spain; 5 Department of Pediatric Hematology, Oncology and Bone Marrow Transplantation, Wroclaw Medical University, Wroclaw, Poland; 6 Pavlov First St. Petersburg State University, Raisa Gorbacheva Memorial Institute for Pediatric Oncology, Hematology and Transplantation, St. Petersburg, Russia; 7 Department of Pediatric Hematology and Oncology, Universitätsklinikum Frankfurt, Frankfurt, Germany; 8 Department of Pediatric Hematology and Oncology, Ospedale S Orsola Malpighi, Bologna, Italy; 9 Department of Pediatrics, Jena University Hospital, Jena, Germany; 10 Department of Pediatric Hematology and Oncology, Universitätsklinikum Münster, Münster, Germany; 11 Department of Pediatric Hematology and Oncology, Universitätsklinikum Tübingen, Tübingen, Germany

**Keywords:** Cancer immunotherapy, Bone metastases

## Abstract

Patients with advanced Ewing sarcoma (AES) carry a poor prognosis. Retrospectively, we analyzed 66 AES patients treated with allogeneic stem cell transplantation (allo-SCT) receiving HLA-mismatched (group A, *n* = 39) versus HLA-matched grafts (group B, *n* = 27). Median age at diagnosis was 13 years, and 15 years (range 3–49 years) at allo-SCT. The two groups did not differ statistically in distribution of gender, age, remission status/number of relapses at allo-SCT, or risk stratum. 9/39 (23%) group A versus 2/27 (7%) group B patients developed severe acute graft versus host disease (GvHD). Of patients alive at day 100, 7/34 (21%) group A versus 9/19 (47%) group B patients had developed chronic GvHD. In group A, 33/39 (85%) versus 20/27 (74%) group B patients died of disease and 1/39 (3%) versus 1/27 (4%) patients died of complications, respectively. Altogether 12/66 (18%) patients survived in CR. Median EFS 24 months after allo-SCT was 20% in both groups, median OS was 27% (group A) versus 17% (group B), respectively. There was no difference in EFS and OS in AES patients transplanted with HLA-mismatched versus HLA-matched graft in univariate and multivariate analyses. In this analysis, CR at allo-SCT is a condition for survival (*p* < 0.02).

## Introduction

Patients with advanced Ewing sarcoma (AES, here defined by the presence of ≥2 bone metastases, and/or bone marrow involvement and/or relapse ≤2 years after diagnosis) carry a poor prognosis, emphasizing the need to identify innovative therapy options for these patients. The success of immunotherapy in the treatment of solid tumors is predominantly restricted to entities showing high numbers of tumor infiltrating T cells directed against somatic mutation derived neo-antigens, such as in melanoma. However, unlike melanoma, pediatric cancers, such as ES, are less immunogenic, probably due to low somatic mutation rates and immunosuppressive behavior.

Eliciting a pro-inflammatory environment following allo-SCT may lead to enhanced phagocytic-, natural killer (NK)—as well as to T cell activity [1–3]. In this regard, there is supporting evidence that allo-reactive donor-NK cell as well as T cells play a role in controlling minimal residual disease. Therefore, These donor lymphocyte infusions (DLI) may reduce relapse rates in leukemia and solid pediatric tumors [4–7].

In the past, reduced-intensity conditioning regimens were implemented to reduce chemotherapy-associated toxicity compared to high-dose chemotherapy conditioning in order to facilitate a presumed graft-versus-tumor effect in patients with refractory ES. However, in a retrospective analysis performed by us, reduced toxicity was replaced by higher relapse rates leading to equal overall survival (OS) compared to high-dose chemotherapy-based regimens. The therapeutic benefit of HLA-mismatched allo-SCT to induce a graft-versus-tumor effect remained unclear due to the small number of haplo-transplanted patients at that time [8].

The present study is an partial update of our analysis on the role of allo-SCT in AES Patients conducted in 2010—now focusing on the use of HLA-mismatched vs. HLA-matched transplants [8]. We hypothesized that the presence of a graft versus tumor effect would improve survival in a subgroup of patients treated with HLA-mismatched allo-SCT versus HLA-matched allo-SCT due to HLA disparity.

## Patients and methods

### Study design and data provenience

We collected and evaluated data of 66 patients treated with allo-SCT due to AES between 2000 and 2015 in pediatric transplantation centers in Germany (*n* = 26), Italy (*n* = 15), Spain (*n* = 6), Russia (*n* = 6), France (*n* = 5), Austria (*n* = 2), Lithuania (*n* = 2), Poland (*n* = 2), Israel (*n* = 1), and Jordan (*n* = 1). Inclusion criteria were presence of AES and allo-SCT treatment after 1999. Diagnosis was based on histopathological examination and in recently diagnosed patients confirmed by molecular-genetic detection of ES specific translocations. In the following sections, patient numbers are followed by specification of respective proportions given in brackets when appropriate, except when data were unavailable.

### Definitions

Engraftment was defined as an absolute neutrophil count ≥0.5 × 10^9^/L after allo-SCT. In case patients died within ≤100 days after allo-SCT or when information was unavailable, chronic graft versus host disease status was considered as not assessable. Death of complications (DOC) constituted any kind of treatment-related death occurring after allo-SCT in the absence of disease evidence, including engraftment failure. In contrast, the definition of death of disease (DOD) comprised any death directly related to either disease progression or relapse. Progressive disease (PD) was defined as ≥50% progression of tumor volume, partial remission (PR) as ≥50% reduction and complete remission (CR) as absence of detectable disease. Residual disease (RD) included both PD and PR according to RECIST criteria v .1.1. Early relapse was defined as relapse occurrence ≤24 months after diagnosis as opposed to the definition of late relapse (>24 months after diagnosis). Multifocal disease was defined as ≥3 involved bone sites and/or bone marrow (BM) involvement at diagnosis. HLA-mismatch was defined as ≤9/10 differing HLA class 1 and class 2 alleles. OS was defined as the period between last allo-SCT and death of any cause or last follow-up. Event free survival (EFS) was defined as the period from last allo-SCT until either relapse or death due to non-CR at allo-SCT and progressive disease and/or DOC.

### Patients

The study population consisted of 42 (0.64) male and 24 (0.36) female patients. Median age at diagnosis was 13 years (range 1–49 years) and median age at allo-SCT was 15 years (range 3–49 years). Before allo-SCT, all patients were assigned either to group A (transplanted with HLA-mismatched grafts) or to group B (transplanted with HLA-matched grafts). Group A comprised 39 and group B comprised 27 patients. Within group A 28/39 patients received a haplo-identical graft, 6/39 received a 2/10 mismatched graft, 2/39 received a 3/10 mismatched graft, and 3/39 received a 4/10 mismatched graft. Eligibility for allo-SCT was decided upon the presence of ≥2 bone metastases and/or bone marrow involvement at diagnosis or at relapse and/or relapse ≤2 years after diagnosis. Altogether, after induction and conditioning treatment 22/66 (33%) patients were transplanted in CR and 44/66 (67%) with RD. Gender, age at diagnosis, remission at allo-SCT, relapse at allo-SCT, BM involvement at diagnosis and risk strata did not differ significantly between both groups. Most patients have been heavily pretreated including multiple transplantations prior to allo-SCT. In group A, 12 patients received one auto-SCT and one allo-SCT, seven patients received two auto-SCT followed by one allo-SCT, two patients received two allo-SCTs, two patients received one auto-SCT followed by two allo-SCTs and 11 patients only received one allo-SCT, respectively. In group B, 14 patients received one auto-SCT and one allo-SCT, four patients received two auto-SCTs followed by one allo-SCT and nine patients received only one allo-SCT, respectively. Altogether, 10/27 group A versus 18/39 group B patients received prior auto-SCT before allo-SCT. In group A, eight patients received bone marrow (BM) derived transplants and 31 patients received peripheral blood derived stem cells (PBSC). In group B, 17 patients received BM and 10 patients received PBSC. In group A, 12/39 patients received primary allo-SCT due to multifocal disease, whereas 13/39, 9/39 and 5/39 patients received allo-SCT due to first, second and third relapse, respectively. In group B, 17/27 patients received primary allo-SCT due to multifocal disease, whereas 6/27 and 2/27 patients received allo-SCT due to first and second relapse. The difference between distribution of primary versus relapse as the cause for allo-SCT eligibility was not significant (*p* = 0.57). Patients’ characteristics are provided in Table 1. Treatment application relied upon institutional review board approvals according to the precepts established by the Helsinki Conference Declaration. All patients or their guardians signed informed consent prior to therapy.

**Table 1:** Patient characteristics.

	Group A (HLA-mismatched)	fraction	Group B (HLA-Matched)	fraction	*p*-value
Total	39		27		
Gender					
m	24	0.62	18	0.67	n.s.
f	15	0.38	8	0.33	
Age at diagnosis					
≤14	27	0.69	13	0.48	n.s.
>14	12	0.31	14	0.52	
Number of allele mismatch (Group A)					
Haploidentical	28		n.a.		n.a.
2/10	6		n.a.		n.a.
3/10	2		n.a.		n.a.
4/10	3		n.a.		n.a.
Eligibility for allo-SCT					
Multifocal/progressive primary disease	12	31%	17	63%	n.s.
Relapse	27	69%	10	37%	
* First relapse*	*13*	*33%*	*6*	*22%*	
* Second relapse*	*9*	*23%*	*2*	*7%*	
* Third relapse*	*5*	*13%*	*0*	*0%*	
* Unknown*	*0*	*0%*	*2*	*7%*	
Remission at allo-SCT					
CR	14	0.36	8	0.3	n.s.
Other	25	0.64	19	0.7	
Risk stratum					
R1 + R2loc	10	0.26	4	0.15	n.s.
R2pulm	5	0.12	4	0.15	
R3 (extrapulm met)	24	0.62	19	0.7	

*m* male, *f* female, *CR* complete remission, *R1* *+* *R2loc* localized disease, *R2pulm* localized disease with pulmonary metastases, *R3(extrapulm met)* ≥2 bone metastases at diagnosis, *allo-SCT* allogeneic stem cell transplantation, *n.s.* not significant.

### Conditioning regimens and GvHD prophylaxis

Conditioning regimen mainly based on the reduced-toxicity use of melphalan (140 mg/m^2^) in combination with fludarabine (120–180 mg/m^2^) and thiotepa (10 mg/kg) as well as anti-thymocyte globuline or OKT3. Furthermore, other regimen containing cyclophosphamide (120 mg/m^2^) busulfan (8 mg/kg) and topotecan (6 mg/m^2^) (Supplementary Table S1). For assessment of conditioning regimens only the effect of the latest allo-SCT was analyzed even in case some patients had received auto- or allografts before. GvHD prophylaxis and treatment included use of methotrexate, mycophenolat-mofetil, cyclosporine A, and/or prednisolone. In haploidentical transplantations, ex vivo graft manipulation with immunomagnetic CD3/CD19 was used as GvHD prophylaxis. Patients with ex vivo manipulated grafts received no GvHD prophylaxis or short course mycophenolate-mofetil.

### Statistical analysis

End points were assessed upon the date of last patient contact. Final data base update was conducted in June 2018. Statistical analyses were performed using R 2.11.0 (The R Foundation for Statistical Computing, Vienna Austria), SAS and SPSS. Time values for DOC and relapse/DOD estimates were assessed starting on the date of the last allo-SCT until last follow-up or relapse and for OS until last follow-up and/or until the occurring event was death independent of the cause. In multivariate analyses, considered variables were graft type (HLA-matched vs. -mismatched), patient age at allo-SCT (grouped ≤14 or >14 years), gender and disease status at allo-SCT. Hazard ratios (HR), standard errors and confidence intervals (CI) are given when appropriate.

For calculation of OS probabilities, the Kaplan–Meier estimate was used. OS curves were compared using the two-tailed log-rank test. Associations of patient characteristics and conditioning regimens with OS were evaluated in multivariate analyses using Cox proportional hazards. A *p* value < 0.05 was considered statistically significant.

## Results

### Engraftment and GvHD

63/66 (95%) patients experienced primary engraftment of neutrophils. Three patients (5%) experienced primary graft failure (defined as the absence of initial donor cell engraftment with donor cells <95%, peripheral blood ANC < 0.5 × 10^9^/L by day+28 after allo-SCT) and experienced successful engraftment following a second allo-SCT. Median neutrophil engraftment was 15 days in group A and 17 days in group B patients. 22/39 (56%) group A versus 13/27 (48%) group B patients developed acute graft versus host disease grade I–IV (GvHD). In group A 25/39 (64%) patients showed no or grade I, 5/39 (13%) had grade II and 9/39 (23%) had grade III. No grade IV GvHD occurred. In group B 14/27 (52%) patients showed no or grade I, 11/27 (41%) had grade II and 1/27 (4%) had grade IV GvHD, respectively. Of patients alive at day 100, 6/34 (18%) and 1/34 (3%) group A patients developed limited or severe chronic GvHD, respectively. In group B, 7/19 (37%) and 2/19 (10%) group B patients developed limited or severe chronic GvHD, respectively. Status was unknown in two patients and not assessable in 11/66 patients. The difference in aGvHD incidence did not differ significantly between both groups. An overview is provided in Table 2.

**Table 2:** Disease course after allo-SCT.

	Group A (HLA-Mismatched *n* = 39)			Group B (HLA-Matched *n* = 27)			
	Number		Fraction	Number		Fraction	*p* value
Outcome							
Engraftment							
Primary	36		92%	27		100%	
Secondary	3		8%	0		0	n.s.
aGvHD							
None or grade I	25		64%	14		52%	n.s.
Grade II	5		13%	11		41%	n.s.
Grade III	9		23%	1		4%	n.s.
Grade IV	0		0%	1		4%	n.s.
cGvHD							
None	26		67%	10		37%	n.s.
Limited	6		15%	7		26%	n.s.
Extensive	1		3%	2		7%	n.s.
N.A. due to death or last FU ≤ d100	5		13%	7		26%	n.s.
Data not available	1		3%	1		4%	n.s
Outcome							
DOC	1		3%	1		4%	n.s
DOD	32		82%	20		74%	n.s
Alive at last FU	6		15%	6		22%	n.s.
2 Years EFS		20%			20%		n.s.
Median EFS (months after allo-SCT)							
Median		7.5			5		n.s.
Median OS (months after allo-SCT)							
Median		8			6		0.04

*aGvHD/cGvHD* acute/chronic graft versus host disease, *OS* overall survival, *DOC* death of complications, *DOD* death of disease, *FU* follow-up, *allo-SCT* allogeneic stem cell transplantation, *N.a.* not assessable, *n.s.* not significant.

### Survival

**Fig. 1:**
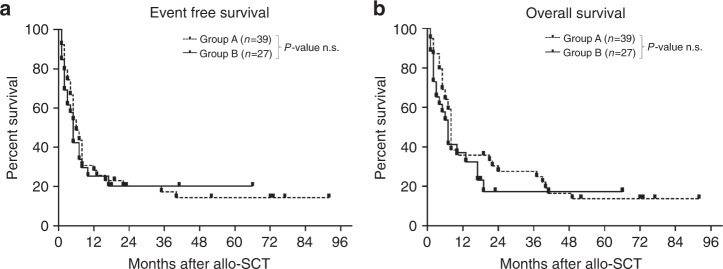
Event free survival and overall survival - Allo-SCT with HLA-mismatched versus HLA-matched grafts. **A** Event-free survival and **B** overall survival probabilities after allogeneic stem cell transplantation (allo-SCT) for patients transplanted with HLA-mismatched grafts (group A; *n* = 39) – versus HLA-matched grafts (group B; *n* = 27); Patients alive at last follow-up were censored. The differences are not significant (Log Rank, *P* > 0.5).

**Fig. 2:**
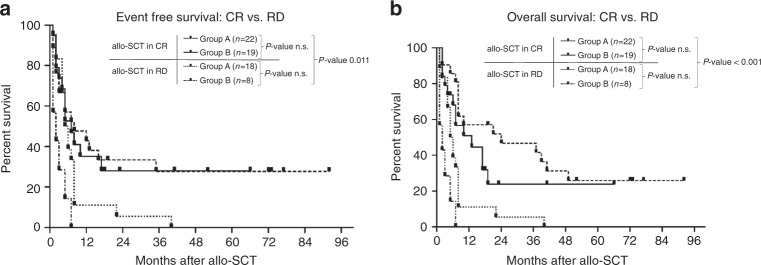
Event free survival and overall survival - Allo-SCT in complete remission versus residual disease. **A** Event-free survival and **B** overall survival after allogeneic stem cell transplantation (allo-SCT) until relapse/death of disease in patients transplanted in complete remission (CR) versus transplantation in residual disease (RD), respectively. Patients alive at last follow-up were censored. With *p* values < 0.05 in both analyses, EFS and OS is significantly higher in patients treated in CR.

In this analysis, time periods from the date of the last allo-SCT until the occurrence of relapse/DOD were compared between both groups. Relapse and progression were the predominant causes of death. Median EFS and OS was 6 and 8 months (group A) and 5 months and 6 months (group B), respectively (Fig. 1A, B). Median EFS at 24 months after allo-SCT was 20% in both groups, median OS was 27% (group A) versus 17% (group B), respectively. Median follow-up was 7.5 months. When both groups were further divided, the respective median EFS and OS differed significantly between groups with CR versus RD at allo-SCT, respectively (*p* = 0.011 and *p* < 0.001, respectively; Fig. 2A, B). Only patients in CR at the time of allo-SCT survived. Difference of mean OS was significantly different between both groups (*p* = 0.04, Welch-test, Table 2). In Kaplan–Meier curves, however, this could not be confirmed. There was no difference in EFS and OS in AES patients transplanted with HLA-mismatched vs. HLA-matched grafts in either setting, indicating that a hypothesized graft-versus-tumor effect is not enhanced in this analysis. Furthermore, in group A patients, the use of haplo-identical- versus other HLA-mismatched grafts had no significant impact on survival (data not shown).

### Causes of death

In group A, 33/39 (0.85) versus 20/27 (0.74) group B patients died of disease progression or relapse and 1/39 (0.03) versus 1/27 (0.04) patients died of complications, respectively. Altogether 12/66 (0.18) patients survived in CR.

### Multivariate analysis

Multivariate analyses confirmed univariate analyses. Only patients reaching CR prior to allo-SCT had a chance to be cured (for EFS; *p* = 0.01, HR 0.4, 95% CI 0.20–0.77, for OS; *p* < 0.01, HR 0.29, 95% CI 0.15–0.57). None of the considered other variables, i.e., graft-type age at allo-SCT, gender and BM involvement had an influence on survival outcome (Table 3).

**Table 3:** Multivariate analysis.

	HR	SE	95% CI	*p* value
EFS				
Age at allo-SCT				
<14years	Reference			
>=14years	0.84	0.45	Jan-58	n.s.
Gender				
Male	Reference			
Female	1.18	0.68	2.06	n.s.
Graft Type				
HLA matched	Reference			
HLA mismatched	0.84	0.45	1.58	n.s.
Disease Stage at allo-SCT				
RD	Reference			
CR	0.4	0.20	0.77	0.01
OS				
Age at allo-SCT	Reference			
	0.76	0.42	Jan-36	n.s.
Gender				
Male	Reference			
Female	1.31	0.74	2.31	n.s.
Graft Type				
HLA matched	Reference			
HLA mismatched	0.76	0.41	1.39	n.s.
Disease Stage at allo-SCT				
RD	Reference			
CR	0.29	0.15	0.57	<0.01

*EFS* event free survival, *OS* overall survival, *DOD* death of disease, *HR*, hazard ratio, *SE* standard error, *CI* confidence Interval, *RD* residual disease, *CR* complete remission, *allo-SCT* allogeneic stem cell transplantation, *n.s.* not significant.

## Discussion

In this work, we hypothesized that HLA-mismatched versus HLA-matched allo-SCT would improve survival in a group of ES patients with advanced disease. We divided the group in those patients who received HLA-matched- and those who received HLA-mismatched grafts. All patients were treated in a time span of 15 years (2000–2015) in order to facilitate comparability. We hypothesized that in this setting it would be possible to control tumor growth, in particular minimal residual disease, due to HLA disparity in HLA-mismatched—versus HLA matched transplantation settings.

In a recent analysis, the INFORM consortium analyzed genetic alterations in 961 tumors from children, adolescents and young adults. The conclusion was, that genetic alterations in 149 putative cancer driver genes may separate tumors in two classes: small mutation and structural/copy-number variants [9]. The rationale to conduct immunotherapeutic approaches in non-immunogenic tumors, such as it is supposedly the case in most pediatric tumors [9, 10], lies in the induction of an inflammatory microenvironment rendering cancer susceptible to an immunotherapeutic attack. The addition of chemotherapy, hyperthermia or irradiation may result in tissue injury, tumor destruction and in the secretion of pro-inflammatory cytokines [2]. Thereby, antigen presentation is augmented via mechanisms such as antigen cross-presentation, HLA up-regulation of tumor or stromal cells leading to enhanced donor TCR-mediated T cell recognition and further activation of the transplanted adaptive immune system via cytokines (e.g., IL-2, TNF, and IFNg) and higher expression of co-stimulatory molecules (e.g., CD40/CD40L or B7/CD28) [2, 11]. Merchant et al. observed improved overall survival in ES patients treated with autologous T cells in combination with tumor-lysate pulsed dendritic cells and IL-7 [12]. Interestingly, the use of histone-deacetylase (HDAC)-inhibitors seem to enhance activity of cancer specific central memory T cells in solid tumors, possibly enhancing immune responses [13]. The role of HDAC-inhibitors to augment immunotherapeutic responses against ES, e.g., by up-regulating immune checkpoint inhibitors is subject to current investigation in the *Individualized Therapy For Relapsed Malignancies in Childhood* (INFORM) trials [14].

In past approaches, reduced-intensity conditioning regimens were implemented to reduce chemotherapy-associated toxicity compared to high-dose chemotherapy conditioning in order to facilitate a graft-versus-tumor effect in patients with refractory ES. Baird et al. described long-term overall survival of 3/11 patients with metastasized ES after allo-SCT [15]. These results did not exceed the results of standard treatment for patients with the same risk profile [16]. In a further study we demonstrated that unspecific DLIs after allo-SCT are associated with tumor control in AES and stage IV rhabdomyosarcoma patients [17]. This effect is bought with the risk for life-threatening GvHD, emphasizing the need to identify tumor-specific e.g., cellular therapeutic approaches.

In a retrospective analysis performed by us, reduced toxicity was replaced by higher relapse rates leading to equal OS compared to high-dose chemotherapy-based regimens. In this analysis the therapeutic benefit of allo-SCT to induce a graft-versus-tumor effect remained unclear [8]. The latter analysis confirmed former results described by Burdach et al. [18]. However, in our analysis performed in 2011, we did not sufficiently address the role of haplo-difference to induce a graft-versus-ES effect after allo-SCT [8]. This was due to the fact, that back then haplo-SCT was performed to a far lesser extent compared to HLA-matched allo-SCT due to the elevated risk of potentially life threatening GvHD. This has changed during the past 10 years due to improvements in ex vivo graft manipulation for GvHD control.

In haploidentical transplantations, ex vivo graft manipulation with immunomagnetic CD3/CD19 was used as GvHD prophylaxis, which may have an influence of a presumed graft versus tumor effect. However, in the present analysis, GvHD rates were comparable. In comparison to patients with leukemia who received T/B cell depleted grafts resulting in only 7% grade III–IV GvHD [19], the incidence in our mismatched group was increased. Reasons might be the relatively low number of patients and the fact, that we also included patients with grafts from mismatched unrelated (non-haploidentical) donors who received no ex vivo T/B cell depletion. Interestingly, despite even the use of mismatched grafts, DOC rates were very low, which is comparable to data provided by Llosa et al. [20]. Almost all patients died of disease. None of the patients with residual disease at the time of allo-SCT survived, identifying CR at allo-SCT as a condition for survival. A stable EFS of ~30% could be reached in these patients. There was no difference in EFS and OS in AES patients transplanted with HLA-mismatched vs. HLA-matched grafts. An allo-immune effect is per se not tumor specific, as seen in the incidence of GvHD. Our hypothesized presence of a graft versus AES effect was clinically not relevant, but could not be excluded in this setting. HLA mismatched transplantation with parental donors, however, may constitute an option for potential immunotherapeutic approaches post-transplant. Of note, the outcome in allo-SCT treated patients was comparable to autologous-SCT in relapsed patients [21]. Patients with multifocal/high risk primary disease versus relapsed disease (including multiple relapses) was 31%/69% in group A and the opposite 63%/37% in group B. Thus, selection bias is not excluded as group A may have had higher treatment difficulties due to a supposed higher risk profile, which may have abrogated a difference in the outcomes between both groups. In conclusion, this retrospective data has to be verified in prospective studies. It will be subject to future investigation to determine whether allo-SCT implicates a therapeutic benefit over standard therapy in patients with advanced ES.

## Funding

The work was funded by grants to Stefan Burdach and Uwe Thiel from the Wilhelm Sander-Stiftung (2018.072.1) as well as Cura Placida. Furthermore Uta Dirksen received grants from the German Cancer AID 102802, 70112018, 70113419.

## Supplementary information

Supplemental Table 1
