# Correction to: No difference in survival after HLA mismatched versus HLA matched allogeneic stem cell transplantation in Ewing sarcoma patients with advanced disease

**DOI:** 10.1038/s41409-021-01421-8

**Published:** 2021-08-09

**Authors:** U. Thiel, S. J. Schober, A. Ranft, H. Gassmann, S. Jabar, K. Gall, I. von Lüttichau, A. Wawer, E. Koscielniak, M. A. Diaz, M. Ussowicz, I. Kazantsev, B. Afanasyev, M. Merker, T. Klingebiel, A. Prete, B. Gruhn, P. Bader, H. Jürgens, U. Dirksen, R. Handgretinger, S. Burdach, P. Lang

**Affiliations:** 1 Department of Pediatrics and Children’s Cancer Research Center, School of Medicine, Kinderklinik München Schwabing, Technical University of Munich, Munich, Germany; 2 Pediatrics III, West German Cancer Centre Essen, University Hospital Essen, Essen, Germany; 3 Department of Pediatric Oncology, Hematology and Immunology, Olgahospital, Stuttgart, Germany; 4 Department of Pediatric Hematology—Oncology and Hematopoietic Stem Cell Transplantation, Hospital Infantil Universitario Niño Jesus, Madrid, Spain; 5 Department of Pediatric Hematology, Oncology and Bone Marrow Transplantation, Wroclaw Medical University, Wroclaw, Poland; 6 Raisa Gorbacheva Memorial Institute for Pediatric Oncology, Hematology and Transplantat, Pavlov First St. Petersburg State Universityion, St. Petersburg, Russia; 7 Department of Pediatric Hematology and Oncology, Universitätsklinikum Frankfurt, Frankfurt, Germany; 8 Department of Pediatric Hematology and Oncology, Ospedale S Orsola Malpighi, Bologna, Italy; 9 Department of Pediatrics, Jena University Hospital, Jena, Germany; 10 Department of Pediatric Hematology and Oncology, Universitätsklinikum Münster, Münster, Germany; 11 Department of Pediatric Hematology and Oncology, Universitätsklinikum Tübingen, Tübingen, Germany

**Keywords:** Cancer immunotherapy, Bone metastases

Correction to: *Bone Marrow Transplantation* (2021) **56**:1550–1557; 10.1038/s41409-020-01200-x, published online 29 January 2021

Due to a processing error, the shared first authorship between Sebastian Schober and Uwe Thiel nor the shared last authorship between Stefan Burdach and Peter Lang was not taken.

The original article has been corrected.

